# Pressurized carbon dioxide lavage reduces the incidence of a radiolucent line around the tibial component two years after total knee arthroplasty

**DOI:** 10.1186/s13018-022-03204-3

**Published:** 2022-07-15

**Authors:** Ryo Sasaki, Masaki Nagashima, Toshiro Otani, Yoshifumi Okada, Noriyuki Aibara, Kenichiro Takeshima, Ken Ishii

**Affiliations:** 1 Department of Orthopaedic Surgery, School of Medicine, International University of Health and Welfare, 4-3 Kōzunomori, Narita City, Chiba 286-8686 Japan; 2 Department of Orthopaedic Surgery, International University of Health and Welfare Mita Hospital, 1-4-3 Mita, Minato-ku, Tokyo, 108-8329 Japan; 3 Department of Orthopaedic Surgery, International University of Health and Welfare Narita Hospital, 852 Hatakeda, Narita City, Chiba 286-8520 Japan; 4 Department of Orthopaedic Surgery, International University of Health and Welfare Ichikawa Hospital, 6-1-14 Kōnodai, Ichikawa City, Chiba 272-0827 Japan

**Keywords:** Total knee arthroplasty, Pressurized carbon dioxide lavage, Radiolucent line, Aseptic loosening

## Abstract

**Introduction:**

In cemented total knee arthroplasty (TKA), pressurized carbon dioxide (CO_2_) lavage prior to cement fixation can eliminate debris at the bone-cement interface and is considered effective for increasing cement penetration and preventing aseptic loosening. Regarding the risk of a preliminary diagnosis of implant loosening, a radiolucent line (RLL) is a valuable sign. The purpose of this study was to compare the incidence of a tibial RLL at 2 years after TKA with and without pressurized CO_2_ lavage.

**Methods:**

This is a retrospective study. One hundred knees from 98 patients were enrolled in this study. TKA was performed without pressurized CO_2_ lavage (CO_2_− group) for the first 47 knees, and with pressurized CO_2_ lavage (CO_2_+ group) for the next 53 knees. The depth of cement penetration was measured just after surgery, and the incidence of tibial RLL > 2 mm at 2 years after TKA was determined.

**Results:**

Significant differences between groups were not seen regarding pre- and postoperative clinical factors. The depth of cement penetration in each area was significantly higher in the CO_2_+ group. The frequency of knees with RLL > 2 mm was significantly lower in the CO_2_+ group than in the CO_2_− group (*p* < 0.001).

**Conclusions:**

Pressurized CO_2_ lavage improved cement penetration and decreased the incidence of tibial RLL > 2 mm at 2 years after TKA.

## Introduction

Total knee arthroplasty (TKA) is a very effective and safe treatment option for advanced knee osteoarthritis [1], but problems with implant durability remain. As reported in the major arthroplasty registries, the revision risk at 10 years is approximately 5% [2]. In particular, reports have identified aseptic loosening as the most common cause of revision [3, 4]. Reducing aseptic loosening is therefore an important issue for achieving successful clinical outcomes after TKA.

One cause of aseptic loosening of a prosthesis in cemented TKAs involves the interplay of debris at the bone cement interface and low cement penetration [5]. Pressurized carbon dioxide (CO_2_) lavage is considered effective for addressing both of these problems. Previous studies have suggested that removing debris by pressurized CO_2_ lavage increases the cement penetration rate [6], and increased cement penetration depth improves the durability of the bone cement interface [7]. For these reasons, pressurized CO_2_ lavage has been expected to increase the cement penetration rate and consequently could prevent aseptic loosening of the TKA.

Although a radiolucent line (RLL) at the bone implant interface of 1 mm on radiographs usually develops in the first year postoperatively and does not progress over the longer term [8], RLL > 2 mm has been reported as a valuable sign for preliminary diagnosis of implant loosening, even without revision [8–10]. Reducing the incidence of RLL > 2 mm is therefore considered to reduce the incidence of aseptic loosening. However, to the best of our knowledge, few investigations have evaluated the relationship between the use of pressurized CO_2_ lavage and the incidence of an RLL. The purpose of this study was to compare the incidence of RLL > 2 mm at the tibia bone-implant interface at 2 years after cemented TKA between treatments with and without pressurized CO_2_ lavage. We hypothesized that TKA with pressurized CO_2_ lavage would lead to a lower frequency of RLL after primary TKA.

## Materials and methods

### Subjects

**Fig. 1:**
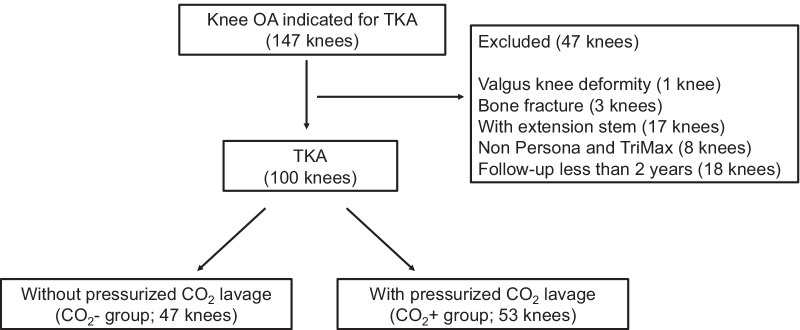
Flowchart of patient recruitment

This is a retrospective study. A total of 147 knee joints in 145 consecutive patients (121 women, 24 men) with knee osteoarthritis underwent cemented TKA at our hospital between April 2015 and March 2018 and were initially considered eligible for inclusion in the present study. Exclusion criteria comprised valgus knee deformity with femorotibial angle (FTA) < 170° (1 knee in 1 patient), bone fracture during follow-up (femoral neck fracture; 3 knees in 3 patients), use of a tibial extension stem (17 knees in 14 patients), use of implants other than Persona (Zimmer Biomet, Warsaw, IN) or TriMax (Ortho Development, Draper, UT) (8 knees in 5 patients), or follow-up < 2 years (18 knees in 18 patients). As a result, 100 knees from 98 patients were enrolled in this study. Forty-seven knees from the first 46 patients underwent TKA without pressurized CO_2_ lavage (CO_2_− group), and 53 knees from the next 52 patients from September 2016 underwent TKA with pressurized CO_2_ lavage (CO_2_+ group) (Fig. 1). This study was approved by the institutional review board, and written informed consent was obtained from all patients.

### Surgical procedures

**Fig. 2:**
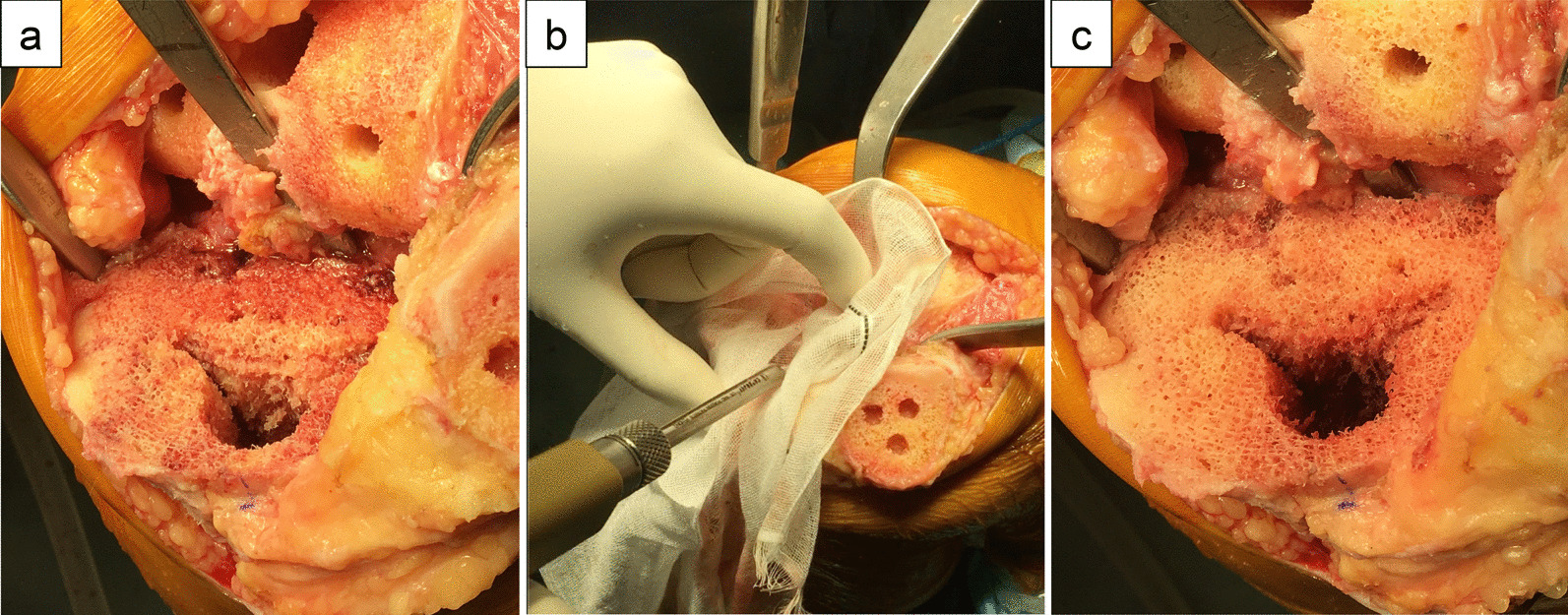
Procedure for using pressurized carbon dioxide (CO_2_) lavage. **a** Debris remains on the bone surface. **b** Pressurized CO_2_ lavage removes the debris. **c** Condition of the bone surface after removing debris, before cement fixation

All TKAs were performed under general anesthesia with tourniquet use, using a measured resection technique aiming for neutral alignment of the knee. The medial parapatellar approach was used. Distal femoral cutting was performed at a valgus angle of 6°–7° using an intramedullary alignment guide. Rotation of the femoral component comprised 3°–5° of external rotation from the posterior condylar axis, aiming at the surgical epicondylar axis. Proximal tibial cutting was performed with an extramedullary guide. Rotation of the tibial component was indexed to the Akagi line [11]. For patellar resurfacing, the thickness of bone resection was taken as the thickness of the patellar component to be placed. The patellar component was medialized on the patellar resected surface, and the component with the largest diameter covering the whole medial aspect of the patella was selected. After placing the trial implant on the patella, lateral patellar facetectomy was performed by bone saw for the lateral aspect of the patella that was not covered by the implant [12]. All components were fixed with bone cement. In both groups, pulsed lavage was applied to remove debris as far as possible before cementing the components [8]. In the CO_2_+ group, debris was additionally removed with pressurized CO_2_ lavage (CarboJet, Kinamed, CA) for about 1 min until micro-bone fragments and fat droplets disappeared just before cementing (Fig. 2). A closed-suction drain was installed and left in the knee for 24 h in all cases. The operation was performed by 7 experienced surgeons (3 in CO_2_+ group, 2 in CO_2_− group, and 2 in both groups). In this study, a fixed-bearing posterior cruciate-stabilizing (PS) or posterior cruciate-retaining (CR) prosthesis was used. In the CO_2_− group, the Persona PS was used in 12 knees, Persona CR in 29 knees, and TriMax PS in 6 knees. In the CO_2_+ group, the Persona PS was used in 26 knees, Persona CR in 8 knees, and TriMax PS in 19 knees.

### Postoperative care

Full-weight walking and range of motion exercises were started the day after surgery in accordance with pain tolerance. For postoperative pain control, a continuous femoral nerve block delivering 0.1% ropivacaine at 4 ml/h was used for 24 h, and oral celecoxib was administered at 200 mg twice daily for 2 weeks after TKA. If pain control was inadequate, a diclofenac sodium suppository (50 mg) and/or intravenous acetaminophen (1000 mg) was used, as appropriate. Although no patients received pharmacological prophylaxis to prevent deep vein thrombosis, intermittent pneumatic compression was used for both feet for one day, and compression stockings were used until the patient was able to walk. In the CO_2_− group, 10 knees were treated for osteoporosis (alfacalcidol or eldecalcitol, 7 knees; bisphosphonate, 3 knees) during follow-up. In the CO_2_+ group, 12 knees were treated for osteoporosis (alfacalcidol or eldecalcitol, 5 knees; bisphosphonate, 7 knees).

### Clinical evaluation

Preoperative medical records were reviewed to obtain information including age, sex, body mass index, range of motion (ROM) of the knee, and Knee Society (KS) Knee Score and Function Score [13]. At 2 years after TKA, ROM of the knee, and KS Knee Score and Function Score were examined. In addition, postoperative complications such as infection were investigated.

### Radiological evaluations

**Fig. 3:**
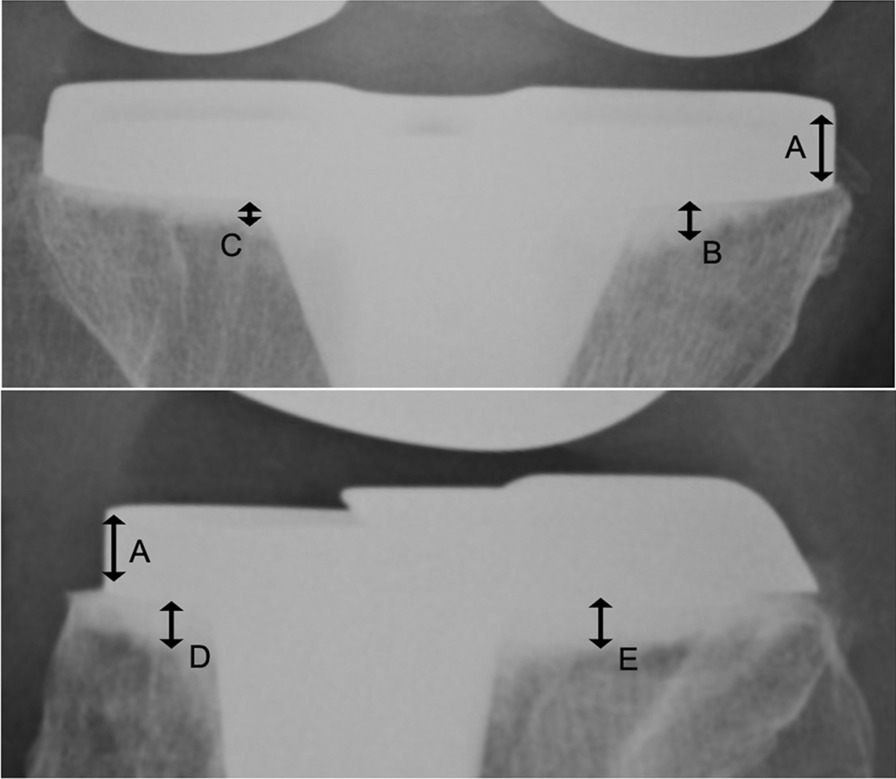
Methods for measurement of cement penetration. Measurements of cement penetration were collected on digital radiographs with a digital ruler calibrated to the thickness of each tibial baseplate (7 mm) (**A**), which was identical for all sizes of this particular implant. The maximum value of cement penetration was measured for each of the medial** (B)**, lateral** (C)**, anterior** (D)**, and posterior** (E)** baseplate zones as defined by the Knee Society Radiographic Evaluation System

**Fig. 4:**
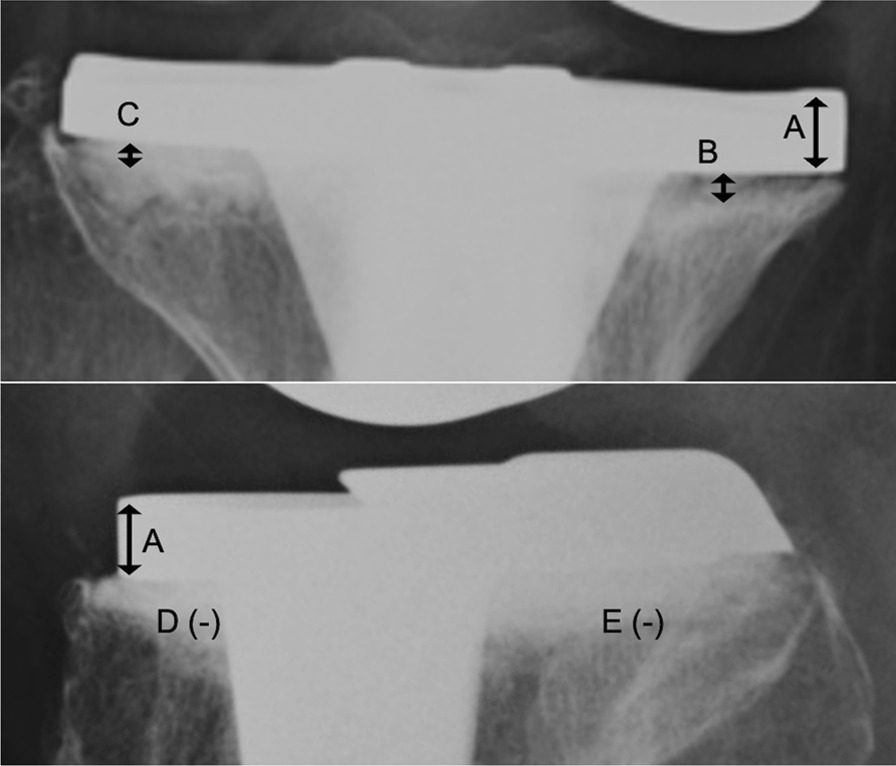
Methods for measurement of radiolucent line (RLL).Measurements of RLL were collected from digital radiographs with a digital ruler calibrated to the thickness of each tibial baseplate (7 mm) (**A**), which was identical for all sizes of this particular implant.The RLL was measured for each of the medial (**B**), lateral (**C**), anterior (**D**), and posterior (**E**) baseplate zones, as defined by the Knee Society Radiographic Evaluation System. The maximum value of all measurements was adopted as the RLL for each case. In this case, the value of (**B**) was adopted as the RLL

For all patients, standardized anteroposterior (AP), lateral, Merchant, and standing whole-leg AP radiographs of the knee were taken before surgery, and the AP, lateral, and Merchant radiographs were taken again at 1, 3, 6, and 12 months after surgery and annually thereafter. Radiographs taken before surgery, just after surgery, and at 2 years after surgery were evaluated in this study. The FTA was measured before surgery. KS radiographic evaluations measured just after surgery included α, β, γ, and Φ [14], and cement penetration around the tibial component. The depth of cement penetration was defined in this study as the maximum depth of cement penetration for each of the medial, lateral, anterior, and posterior baseplate zones of the tibial component (Fig. 3). At 2 years after TKA, the RLL of the tibia was measured. The definition of the RLL and the measurement method were the same as in previous reports [8, 10, 15]. The RLL was measured for each of the medial, lateral, anterior, and posterior baseplate zones defined by the KS Radiographic Evaluation System [14], and the maximum value was adopted as the RLL of the case (Fig. 4). Measurements of cement penetration and RLL were collected on digital radiographs using a digital ruler calibrated to the thickness of each tibial baseplate (7 mm) which was identical for all sizes of this particular implant. The reliabilities of measurements of cement penetrations and RLL were assessed using intraclass correlation coefficients (ICCs). Two investigators interpreted the same radiographs of 20 randomly selected patients. The ICCs for inter- and intra-observer reliabilities were 0.92 and 0.85 for cement penetration and 0.89 and 0.87 for RLL.

### Outcomes

The primary outcome in this study was the incidence of tibial RLL > 2 mm at 2 years after TKA. The secondary outcome was the depth of cement penetration measured just after surgery.

### Statistical analysis

Primary and secondary outcomes and clinical and surgical factors were compared between the two groups using Student’s *t* test, the Mann–Whitney *U* test, and the *χ*^2^ test. Values of *p* < 0.05 were considered significant. Statistical analysis was performed using R version 4.0.3 (R Foundation for Statistical Computing, Vienna, Austria).

## Results

No significant differences between groups were seen regarding preoperative demographic, clinical, or radiographic factors (Table 1). Just after surgery, although KS radiographic evaluations did not differ significantly between groups, the depth of cement penetration in each area was significantly higher in the CO_2_+ group (Table 2). Two years after surgery, mean RLLs in all areas were significantly lower in the CO_2_+ group than in the CO_2_− group. The incidence of knees with RLL > 2 mm was significantly lower in the CO_2_+ group than in the CO_2_− group (*p* < 0.001). Comparing individual baseplate zones, in the medial and anterior areas, RLL > 2 mm was significantly less frequent in the CO_2_+ group than in the CO_2_− group (*p* < 0.001 and *p* < 0.05, respectively). No other clinical factors differed significantly between groups (Table 3). No postoperative complications requiring re-operation were encountered.

**Table 1:** Baseline clinical factors prior to TKA

	CO_2_− group (47 knees)	CO_2_+ group (53 knees)	*p* value
Age (years)	75.4 ± 7.8	74.6 ± 6.8	n.s.
Sex (male/female) (knees)	42/5	48/5	n.s.
BMI (kg/m^2^)	23.8 ± 3.1	22.3 ± 2.3	n.s.
FTA (°)	183.2 ± 9.1	183.7 ± 6.1	n.s.
Extension (°)	− 6.8 ± 6.7	− 7.2 ± 5.9	n.s.
Flexion (°)	122.0 ± 14.7	126.4 ± 12.3	n.s.
KS knee score	34.3 ± 15.3	32.1 ± 12.4	n.s.
KS function score	41.9 ± 10.9	39.3 ± 10.5	n.s.

*n.s.* not significant, *BMI* body mass index, *FTA* femorotibial angle, *KS* Knee Society

**Table 2:** Radiological evaluations just after TKA

	CO_2_− group (47 knees)	CO_2_+ group (53 knees)	*p* value
Implant alignment (°)			
α	96.9 ± 2.3	96.0 ± 1.9	n.s.
β	88.6 ± 1.3	88.6 ± 1.4	n.s.
γ	3.0 ± 2.0	2.5 ± 1.7	n.s.
Φ	83.2 ± 2.5	84.0 ± 2.5	n.s.
Cement penetration of baseplate zones (mm)			
Medial	1.7 ± 0.8	3.5 ± 1.5	< 0.001
Lateral	1.7 ± 0.9	3.2 ± 1.6	< 0.001
Anterior	2.0 ± 1.0	3.0 ± 1.0	< 0.001
Posterior	1.7 ± 0.7	3.2 ± 1.3	< 0.001

*n.s.* not significant

**Table 3:** Clinical and radiological evaluations at 2 years after TKA

	CO_2_− group (47 knees)	CO_2_+ group (53 knees)	*p* value
Extension (°)	− 0.4 ± 1.8	− 0.6 ± 1.6	n.s.
Flexion (°)	118.9 ± 9.0	120.5 ± 8.2	n.s.
KS knee score	91.4 ± 3.8	91.5 ± 2.3	n.s.
KS function score	80.2 ± 12.7	81.8 ± 10.9	n.s.
RLL measurement (mm)			
Medial	1.1 ± 1.0	0.5 ± 0.6	< 0.001
Lateral	0.7 ± 1.0	0.2 ± 0.5	0.004
Anterior	0.5 ± 0.7	0.1 ± 0.3	< 0.001
Posterior	0.4 ± 0.7	0.0 ± 0.2	< 0.001
RLL > 2 mm (knees)			
Medial	19	2	< 0.001
Lateral	11	0	0.006
Anterior	5	0	0.048
Posterior	3	0	n.s.
RLL >2 mm (knees) (%)	18 (38.3%)	2 (3.8%)	< 0.001

*n.s.* not significant, *RLL* radiolucent line, *KS* Knee Society

## Discussion

The present results supported the hypothesis that TKA with pressurized CO_2_ lavage contributes to a reduced frequency of RLL > 2 mm around the tibial component after primary TKA. In this study, although no significant difference in implant placement angles of the femur or tibia were evident between groups, cement penetration just after surgery was significantly increased and the incidence of RLL > 2 mm at 2 years after TKA was significantly reduced in the CO_2_+ group. Since no significant differences were evident in pre- or postoperative clinical parameters between groups, the frequency of the RLL was not associated with clinical outcomes.

The frequency of revision TKA is relatively low in general, but the percentage continues to steadily increase annually [16]. Previous reports have indicated aseptic prosthetic loosening, infection, and postoperative instability as the three most common reasons for all contemporary revision TKA procedures. Furthermore, aseptic loosening was suggested as the cause of revision in 16.1–41.5% of cases over 2 years [5].

Aseptic loosening is a complex reaction thought to be driven by a chronic immune activation that leads to osteolysis [17]. The probability of developing an osteolytic response is likely to involve the combination of environmental and genetic factors [18]. Environmental factors and genetic susceptibilities may trigger an immune response at the implant site, resulting in implant-induced osteolysis [19]. Environmental factors that can lead to aseptic loosening include implant material, component malalignment, high patient weight and activity, low bone quality, and relevant medical histories, such as diabetes and renal failure, with debris at the bone cement interface being one important factor [20].

Pressurized CO_2_ lavage eliminates debris at the bone cement interface and increases cement penetration, and has been considered effective for preventing aseptic loosening [6, 7]. However, the relationship between cement penetration and aseptic loosening remains controversial. On the other hand, RLL > 2 mm has been considered valuable for preliminarily diagnosing loosening of the TKA [8, 9]. Furthermore, previous reports have demonstrated a positive correlation between RLL > 2 mm and the incidence of revision [10]. Reducing the incidence of RLL > 2 mm is thus considered to reduce the incidence of subsequent loosening. However, to the best of our knowledge, no research has evaluated the relationship between cement penetration improved by the use of pressurized CO_2_ lavage and the incidence of RLL > 2 mm. This is the first report to investigate the relationship between the incidence of RLL > 2 mm at 2 years after TKA and the presence or absence of the use of pressurized CO_2_ lavage. The use of pressurized CO_2_ lavage prior to cement fixation was demonstrated to improve cement penetration, similar to previous reports [6], and significantly reduced the incidence of RLL. The use of pressurized CO_2_ lavage can thus be expected to reduce aseptic loosening. The use of pressurized CO_2_ lavage is considered to represent a simple, effective, non-invasive, and reliable method that surgeons can apply. Based on the present results, pressurized CO_2_ lavage should be used as much as possible in TKA and other surgeries requiring cement fixation.

Several limitations to this study need to be considered when interpreting the results. First, this was a retrospective study that included only a small number of patients. A further prospective study or randomized controlled trial including more cases is needed to validate our findings. Second, postoperative computed tomography was not performed, and the rotations of the femoral and tibial components were not compared. Third, the prosthesis and type of prosthesis, such as PS or CR, used in this study were not entirely the same in both groups. However, a previous study reported that whether the prosthesis was PS or CR was not associated with the frequency of implant loosening [21]. Fourth, the management of osteoporosis was not standardized between groups. The management of osteoporosis has been reported to cause differences in the long-term outcomes of TKA [22].

## Conclusions

The use of pressurized CO_2_ lavage prior to cement fixation improved cement penetration just after surgery and decreased the incidence of RLL > 2 mm around the tibial component at 2 years after TKA.

## Data Availability

The datasets for the present study are available from the corresponding author upon reasonable request.
